# Serum levels of leptin and high molecular weight adiponectin are inversely associated with radiographic spinal progression in patients with ankylosing spondylitis: results from the ENRADAS trial

**DOI:** 10.1186/s13075-017-1350-9

**Published:** 2017-06-15

**Authors:** Agnes Hartl, Joachim Sieper, Uta Syrbe, Joachim Listing, Kay-Geert Hermann, Martin Rudwaleit, Denis Poddubnyy

**Affiliations:** 1 Department of Gastroenterology, Infectiology and Rheumatology, Charité Universitätsmedizin Berlin, Hindenburgdamm 30, 12203 Berlin, Germany; 2 German Rheumatism Research Centre, Charitéplatz 1, 10117 Berlin, Germany; 3 Department of Radiology, Charité Universitätsmedizin Berlin, Charitéplatz 1, 10117 Berlin, Germany; 4 Klinikum Bielefeld Rosenhöhe, An der Rosenhöhe 27, 33647 Bielefeld, Germany

**Keywords:** Axial spondyloarthritis, Ankylosing spondylitis, Leptin, Adiponectin, Adipokine, Radiographic progression, Syndesmophytes

## Abstract

**Background:**

Previous research indicates a role of adipokines in inflammation and osteogenesis. Hence adipokines might also have a pathophysiological role in inflammation and new bone formation in patients with ankylosing spondylitis (AS). The aim of this study was to investigate the role of adipokine serum levels as predictors of radiographic spinal progression in patients with AS.

**Methods:**

A total of 120 patients with definite AS who completed a﻿ 2-year follow up in the ENRADAS trial were included in the current study. Radiographic spinal progression was defined as: (1) worsening of the modified Stoke Ankylosing Spondylitis spine (mSASSS) score by ≥2 points and/or (2) new syndesmophyte formation or progression of existing syndesmophytes after 2 years. Serum levels of adipokines (adiponectin (APN) and its high molecular weight form (HMW-APN), chemerin, leptin, lipocalin-2, omentin, resistin, visfatin) were measured using enzyme-linked immunosorbent assays.

**Results:**

There was a significant association between radiographic spinal progression and both leptin and HMW-APN. Baseline serum levels of both adipokines were lower in patients who showed radiographic spinal progression after 2 years. This association was especially evident in men; they had generally lower leptin and HMW-APN serum levels as compared to women. The inverse association between adipokines and radiographic spinal progression was confirmed in the logistic regression analysis: the odds ratios (OR) for the outcome “no mSASSS progression ≥2 points” were 1.16 (95% CI 1.03 to 1.29) and 1.17 (95% CI 0.99 to 1.38), for leptin and HMW-APN, respectively; for “no syndesmophyte formation/progression” the respective OR were 1.29 (95% CI 1.11 to 1.50) and 1.18 (95% CI 0.98 to 1.42), adjusted for the presence of syndesmophytes at baseline, C-reactive protein at baseline, sex, body mass index (BMI), non-steroidal anti-inflammatory drugs intake score over 2 years, and smoking status at baseline.

**Conclusion:**

Serum leptin and HMW-APN predict protection from spinal radiographic progression in patients with AS. Women generally have higher leptin and HMW-APN serum levels that might explain why they have less structural damage in the spine as compared to male patients with AS.

**Trial registration:**

EudraCT: 2007-007637-39. ClinicalTrials.gov, NCT00715091. Registered on 14 July 2008.

## Background

Ankylosing spondylitis (AS) is a chronic inflammatory disease belonging to a family of spondyloarthritides (SpA), which is characterized by inflammation in the pelvis and the spine with subsequent new bone formation that might lead to partial or total ankylosis of the spine. New bone formation in the spine, and particularly development of specific bony bridges between the vertebral bodies, is usually assessed on spinal radiographs and is referred to as radiographic spinal progression. It has been shown in the past that radiographic spinal progression and disease activity are two main determinants of spinal mobility and functional status in AS [1–3]. However, there is substantial individual variation in the radiographic spinal progression rates in AS [4, 5]. It has been shown that baseline syndesmophytes [4, 5], inflammatory activity as assessed by C-reactive protein (CRP), by the Ankylosing Spondylitis Disease Activity Score (ASDAS), or by the presence of inflammatory changes on magnetic resonance imaging (MRI) [6–12], and cigarette smoking [13] are factors associated with more rapid radiographic spinal progression. Further, several biomarkers in the blood were found to be positively associated with new bone formation in the spine: the already mentioned CRP [7], matrix-metalloproteinase-3 [14], vascular endothelial growth factor [15], calprotectin [16], and the adipokine, visfatin [17]. Some biomarkers, such as sclerostin [18] and dickkopf 1 [19], have been associated with radiographic spinal progression, suggesting that these molecules might have a protective effect.

Identification of biomarkers related to the development of structural damage in AS helps not only to predict progression (there are currently no proven therapeutic options to retard progression; however, this might, change in the near future), but it also helps us to better understand disease mechanisms. For instance, until now, the reason why men with AS develop more structural damage in the axial skeleton than women has not been explained [5, 20].

Adipokines are biologically active substances, which are synthesized and released by fat tissue. They have a wide range of regulatory functions not only in energy metabolism, but also in inflammation and osteogenesis [21, 22]. The role of adipokines in the development of inflammation and new bone formation in AS, however, is as yet unclear. The aim of the present study was to investigate the association between adipokine serum levels and radiographic spinal progression in patients with AS.

## Methods

### Patients

Altogether 120 patients from the trial, Effects of NSAIDs on Radiographic Damage in AS (ENRADAS), who completed the study per protocol and for whom serum was available, were included in the analysis. Serum samples were not available for two patients who completed the study per protocol, and these patients were excluded, therefore, from the current study. Baseline characteristics of the 120 patients included in this analysis are shown in Table 1. The design of the ENRADAS trial has been described in detail elsewhere [23]. Briefly, ENRADAS was a prospective, randomized, controlled trial aimed at investigation of the influence of non-steroidal anti-inflammatory drugs (NSAIDs) on radiographic spinal progression in patients with AS. Patients were randomly assigned to treatment with diclofenac either continuously or on demand for a period of 2 years. Other NSAIDs in the same equivalent dose were allowed in patients with diclofenac intolerability; however, about 2/3 of the patients remained on diclofenac until the end of year 2. Tumor necrosis factor (TNF)α blockers were not allowed in the trial. There was no difference in radiographic spinal progression between patients who took NSAIDs continuously and those who took them on demand, justifying the decision to pool both treatment groups.

**Table 1:** Baseline characteristics of the included patients from the ENRADAS trial

Parameter	All patients (n = 120)	Continuous (n = 61)	On demand (n = 59)
Age, years	42.9 ± 10.3	40.7 ± 9.7	45.1 ± 10.4
Male patients, *n* (%)	82 (68.3)	43 (70.5)	39 (66.1)
Symptom duration, years	14.8 ± 12.2	12.8 ± 11.4	16.9 ± 12.7
HLA-B27 positive, *n* (%)	108 (90)	54 (88.5)	54 (91.5)
ASDAS-CRP	2.8 ± 0.7	2.7 ± 0.7	2.8 ± 0.7
BASDAI, points NRS (0–10)	4.2 ± 1.5	4.1 ± 1.5	4.2 ± 1.5
BASFI, points NRS (0–10)	3.3 ± 2.2	2.9 ± 2.1	3.7 ± 2.2
CRP, mg/L	10.3 ± 12.1	7.9 ± 7.4	12.6 ± 15.2
CRP > 5 mg/L, *n* (%)	68 (57.1)	33 (55)	35 (59.3)
BASMI, points (0–10)	2.6 ± 2.2	2.2 ± 2.1	3.0 ± 2.3
mSASSS, points	13.8 ± 17.2	11.1 ± 15.6	16.6 ± 18.4
Patients with syndesmophytes at baseline, *n* (%)	68 (56.7)	32 (52.5)	36 (61.0)
BMI, kg/m^2^	27.2 ± 5.3	27.3 ± 5.2	27.2 ± 5.4

Characteristics are presented as mean ± standard deviation unless indicated otherwise. *ASDAS* Ankylosing Spondylitis Disease Activity Score, *BASDAI* Bath Ankylosing Spondylitis Disease Activity Index, *BASFI* Bath Ankylosing Spondylitis Functional Index, *BASMI* Bath Ankylosing Spondylitis Metrology Index, *BMI* body mass index, *CRP* C-reactive protein, *mSASSS* modified Stoke Ankylosing Spondylitis Spine Score

### Assessment of radiographic spinal progression

Radiographs of the lumbar and cervical spine (lateral projection) were obtained at baseline and after a period of 2 years. Radiographs were centrally collected, digitized, anonymized, and subsequently scored by two experienced and calibrated readers (KGH and DP) in a randomly selected and concealed order according to the modified Stoke Ankylosing Spondylitis Spine Score (mSASSS) system [24]. The mean mSASSS was calculated from the two readers’ scores. Radiographic spinal progression was defined as: (1) mSASSS worsening by ≥2 points after 2 years, and/or (2) development of at least one new syndesmophyte or progression of two single syndesmophytes into a bridging syndesmophyte, in the opinion of both readers after 2 years.

### Measurement of adipokine serum levels

Adipokine serum levels were measured at baseline and after 2 years of follow up (week 100) using commercially available enzyme linked immunosorbent assays (ELISA). The following adipokines were selected based on data from the available literature [25, 26] indicating a possible association with bone metabolism and/or inflammation: adiponectin (APN) (BioVendor - Research and Diagnostic Products, Czech Republic) and its high molecular weight form (HMW-APN) (R&D Systems, MN, USA), chemerin (BioVendor - Research and Diagnostic Products), leptin (Invitrogen, CA, USA) lipocalin-2 (R&D Systems), omentin (BioVendor - Research and Diagnostic Products), resistin (Adipogen International, Switzerland), and visfatin (Adipogen International).

### Statistical analysis

First, we analyzed differences in the mean adipokine serum levels between groups with and without radiographic spinal progression (Mann-Whitney *U* test). Adipokines with significantly different baseline serum levels in progressors and non-progressors were further analyzed. Since adipokines are mainly produced by adipose tissue and their serum levels correlate with body mass index (BMI) [25–27], the values were corrected for BMI by calculating an adipokine/BMI ratio that was used in the analysis in addition to the measured serum levels. Further, an HMW-APN/APN ratio reflecting a proportion of a more biologically active form of APN in the total APN [28] was calculated and included in the analysis. For the analysis of the influences of the treatment arm on the change in serum levels of adipokines, we performed the non-parametric Wilcoxon test on the paired measures, and an analysis of covariance (ANCOVA) with adjustment for the baseline adipokine levels. Receiver operating characteristic (ROC) analysis was performed to evaluate the adipokines as predictors of radiographic spinal progression. The association between serum adipokines and radiographic spinal progression was further explored in the logistic regression analysis. The parameter estimates were adjusted for the presence of syndesmophytes at baseline, baseline CRP, sex, BMI (for crude adipokine serum levels and the HMW-APN/APN ratio), NSAID intake score over 2 years, and smoking status at baseline. A *p* value <0.05 was considered to be statistically significant.

## Results

A total of 29 patients had radiographic progression defined as mSASSS worsening by ≥2 points after 2 years; 25 patients had syndesmophyte formation/progression. The intraclass correlation coefficient (ICC) between the two readers of the X-rays was good: the ICC was 0.96 for baseline and 0.95 for year-2 X-rays; the ICC for change in the mSASSS was 0.50 [23].

Adipokine serum levels in patients with and without radiographic spinal progression are presented in Table 2. There were significant differences between progressors and non-progressors in the baseline serum levels of leptin and HMW-APN (and to a further extent in the HMW-APN/APN ratio). Therefore, these adipokines were included in the subsequent analysis.

**Table 2:** Levels of adipokines in patients with AS with and without radiographic spinal progression after 2 years

Adipokine	mSASSS progression ≥2 points (n = 29)	No mSASSS progression ≥2 points (n = 91)	*P* ^a^	Syndesmophyte formation/progression (n = 25)	No new syndesmophyte formation/progression (n = 95)	*P* ^a^
*Baseline*						
Leptin, ng/mL	10.5 ± 9.0	16.4 ± 13.7	0.003	10.2 ± 9.6	16.2 ± 13.4	0.003
APN, μg/mL	10.1 ± 4.8	10.7 ± 4.4	0.366	9.5 ± 3.7	10.8 ± 4.6	0.210
HMW-APN, μg/mL	4.95 ± 3.52	6.35 ± 4.15	0.045	4.53 ± 2.86	6.40 ± 4.22	0.026
Lipocalin-2, ng/mL	308.4 ± 424.8	197.1 ± 188.8	0.668	257.9 ± 361.3	215.1 ± 238.9	0.851
Chemerin, ng/mL	211.5 ± 54.2	222.6 ± 53.6	0.356	223.4 ± 57.8	219 ± 52.9	0.831
Omentin, ng/mL	455.1 ± 168.3	425.8 ± 131.7	0.477	477.8 ± 164.7	421 ± 132.8	0.122
Resistin, ng/mL	37.7 ± 27.9	30.4 ± 25.3	0.098	35.6 ± 25.4	31.3 ± 26.2	0.161
Visfatin, ng/mL	44.7 ± 80.3	42.2 ± 58.9	0.484	39.98 ± 83.4	43.4 ± 58.7	0.151
*Year 2*						
Leptin, ng/mL	13.4 ± 12.8	17.5 ± 16.0	0.044	11.7 ± 9.8	17.7 ± 16.3	0.032
APN, μg/mL	9.1 ± 3.8	11.0 ± 5.9	0.129	8.6 ± 3.6	11.1 ± 5.8	0.029
HMW-APN, μg/mL	5.06 ± 3.31	5.99 ± 3.88	0.185	4.55 ± 2.8	6.08 ± 3.92	0.071
Lipocalin-2, ng/mL	235 ± 229.3	273 ± 311.1	0.762	194.7 ± 160.3	281.8 ± 316.7	0.613
Chemerin, ng/mL	216.2 ± 60.3	213 ± 56.7	0.990	218 ± 61.9	212.6 ± 56.4	0.893
Omentin, ng/mL	444.7 ± 133.6	426.4 ± 134.6	0.359	452 ± 139.9	425.3 ± 132.6	0.268
Resistin, ng/mL	47.4 ± 84.3	62.4 ± 204.8	0.332	49.8 ± 91	61.1 ± 200.4	0.500
Visfatin, ng/mL	43.6 ± 70	42.6 ± 62.3	0.627	43.9 ± 75.2	42.6 ± 61.2	0.499

*AS* ankylosing spondylitis, *APN* adiponectin, *HMW-APN* high molecular weight adiponectin, *mSASSS* modified Stoke Ankylosing Spondylitis Spine Score. ^a^Mann-Whitney *U* test

HMW-APN serum levels did not differ between the two treatment arms (NSAIDs continuously vs. on demand) at baseline and after 2 years of follow up; there was, however, a difference in the leptin levels at baseline (but not at year 2): 13.6 ± 13.2 ng/mL in the continuous intake group vs. 15.5 ± 12.6 ng/mL in the on-demand group, *p* = 0.046. Overall, there was an increase in the leptin level after 2 years in both treatment groups (continuous vs. on-demand NSAIDs); this was statistically significant in the continuous treatment group only: increase of 13.6 ± 13.2 ng/ml to 15.6 ± 15.6 ng/mL, *p* = 0.002. However, in the ANCOVA, there was no statistically significant difference in the change in the leptin serum level: increase of 2.0 ± 4.84 ng/mL in the continuous vs. 1.26 ± 7.43 ng/mL in the on-demand group, *p* = 0.405, adjusted for baseline leptin. Further, there was no significant association between change in leptin and HMW-APN (or the BMI-corrected values) over 2 years and radiographic spinal progression. Treatment had no effect on the levels of other adipokines.

There was no evidence of association between radiographic spinal progression and BMI in this patient population: BMI at baseline was 26.9 ± 4.1 kg/m^2^ vs. 27.4 ± 5.6 kg/m^2^ in patients with and without mSASSS worsening by ≥2 points, respectively, *p* = 0.93, and 28.1 ± 6.6 kg/m^2^ vs. 27.1 ± 4.9 kg/m^2^ in patients with and without syndesmophytes formation respectively, *p* = 0.58.

**Fig. 1:**
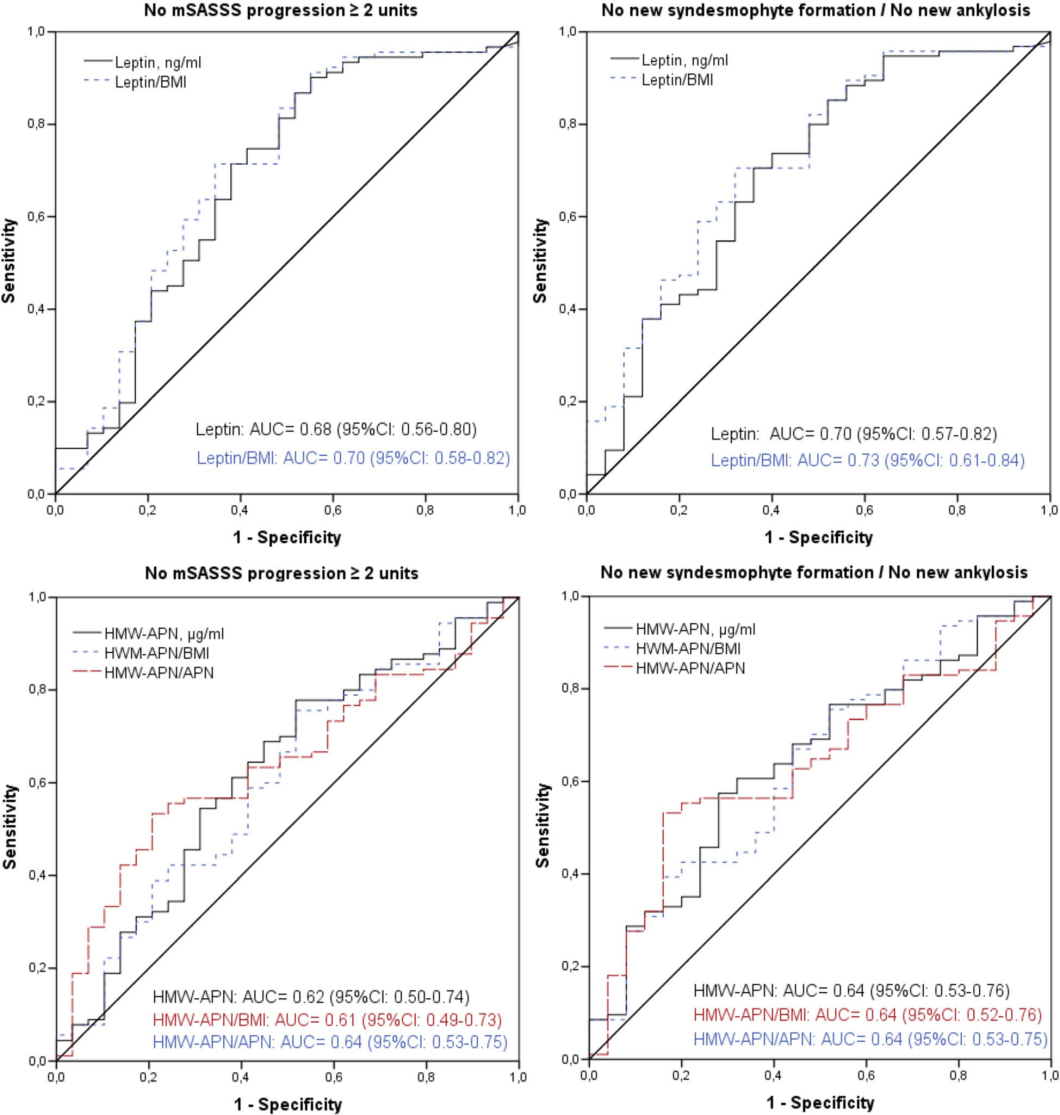
Receiver operating characteristic analysis: association between leptin and high molecular weight adiponectin (*HMW-APN*) serum levels and radiographic spinal progression after 2 years. Baseline serum levels of leptin and HMW-APN shown as crude values and as values corrected for body mass index and adiponectin (only HMW-APN). *mSASSS* modified Stoke Ankylosing Spondylitis Spine Score, *AUC* area under the curve

There was no meaningful correlation between leptin and HMW-APN serum levels, and no correlation with serum CRP. ﻿In the ROC analysis, baseline serum leptin, the leptin/BMI ratio, serum HMW-APN, the HMW-APN/BMI ratio, and the HMW-APN/APN ratio were good predictors of no mSASSS progression ≥2 points and of no syndesmophyte formation/progression (Fig. 1). Leptin and the leptin/BMI ratio was generally a slightly better predictor as compared to HMW-APN.

Remarkably, male patients with AS had significantly lower levels of leptin and HMW-APN (and BMI-adjusted values and HMW-APN/APN ratio) compared to women (Table 3). In the analysis stratified by sex, the association between lower leptin and HMW-APN levels and radiographic spinal progression was evident in men only (Table 4). Furthermore, women had significantly lower mSASSS at baseline and after 2 years of follow up compared to men (7.9 ± 12.6 vs. 16.2 ± 18.2, respectively, *p* = 0.015 at baseline and 8.6 ± 12.9 vs. 17.4 ± 19.0, respectively, *p* = 0.011 after 2 years of follow up).

**Table 3:** Sex-related differences in serum leptin and high molecular weight adiponectin at baseline

	Males (n = 82)	Females (n = 38)	*P* ^a^
Leptin, ng/mL	10.6 ± 6.9	24.4 ± 17.3	<0.001
Leptin/BMI	0.37 ± 0.19	0.84 ± 0.47	<0.001
HMW-APN, μg/mL	5.07 ± 3.06	8.04 ± 5.07	<0.001
HMW-APN/BMI	0.19 ± 0.13	0.32 ± 0.23	0.001
HMW-APN/APN	0.52 ± 0.23	0.61 ± 0.22	0.020

*APN* adiponectin, *HMW-APN* high molecular weight adiponectin, *BMI* body mass index. ^a^Mann-Whitney *U* test

**Table 4:** Baseline serum leptin and HMW-APN in relation to radiographic spinal progression after 2 years

	All patients (n = 120)			Male patients (n = 82)			Female patients (n = 38)		
Outcome: mSASSS progression ≥2 points after 2 years									
	Progressors (n = 29)	Non-progressors (n = 91)	*p* ^a^	Progressors (n = 22)	Non-progressors (n = 60)	*p* ^a^	Progressors (n = 7)	Non-progressors (n = 31)	*p* ^a^
Leptin, ng/mL	10.5 ± 9.0	16.4 ± 13.7	0.003	7.0 ± 4.7	11.9 ± 7.1	0.001	21.7 ± 10.5	25.0 ± 18.6	0.96
Leptin/BMI	0.38 ± 0.31	0.57 ± 0.38	0.001	0.25 ± 0.14	0.42 ± 0.19	<0.001	0.78 ± 0.35	0.86 ± 0.49	0.84
HMW-APN, μg/mL	4.95 ± 3.52	6.35 ± 4.15	0.045	3.93 ± 2.56	5.49 ± 3.15	0.018	8.16 ± 4.37	8.02 ± 5.27	0.75
HMW-APN/BMI	0.19 ± 0.14	0.25 ± 0.19	0.089	0.15 ± 0.11	0.21 ± 0.13	0.052	0.32 ± 0.18	0.33 ± 0.24	0.84
HMW-APN/APN	0.47 ± 0.19	0.57 ± 0.23	0.024	0.42 ± 0.14	0.56 ± 0.24	0.033	0.62 ± 0.25	0.60 ± 0.22	0.82
Outcome: syndesmophyte formation/progression after 2 years									
	Progressors (n = 25)	Non-progressors (n = 95)	*p* ^a^	Progressors (n = 21)	Non-progressors (n = 61)	*p* ^a^	Progressors (n = 4)	Non-progressors (n = 34)	*p* ^a^
Leptin, ng/mL	10.2 ± 9.6	16.2 ± 13.4	0.003	9.0 ± 9.1	11.1 ± 5.9	0.018	16.4 ± 10.9	25.3 ± 17.8	0.27
Leptin/BMI	0.33 ± 0.22	0.57 ± 0.39	0.001	0.29 ± 0.19	0.40 ± 0.18	0.006	0.56 ± 0.23	0.88 ± 0.48	0.13
HMW-APN, μg/mL	4.53 ± 2.86	6.4 ± 4.22	0.026	4.1 ± 2.58	5.4 ± 3.17	0.075	6.8 ± 3.6	8.2 ± 5.23	0.85
HMW-APN/BMI	0.18 ± 0.13	0.25 ± 0.19	0.035	0.16 ± 0.11	0.21 ± 0.13	0.081	0.28 ± 0.18	0.33 ± 0.24	0.81
HMW-APN/APN	0.46 ± 0.2	0.57 ± 0.23	0.033	0.42 ± 0.15	0.55 ± 0.24	0.057	0.68 ± 0.32	0.60 ± 0.21	0.81

*APN* adiponectin, *HMW-APN* high molecular weight adiponectin, *BMI* body mass index, *mSASSS* modified Stoke Ankylosing Spondylitis Spine Score. ^a^Mann-Whitney *U* test

We further explored an association between leptin or HMW-APN and radiographic spinal progression in the logistic regression analysis (Table 5). Serum leptin and the leptin/BMI ratio were significantly inversely associated with radiographic spinal progression; this remained significant after adjusting for factors considered as confounders. The same trend was observed for HMW-APN (crude and BMI-adjusted levels), but this was not statistically significant. However, the HMW-APN/APN ratio was inversely associated with radiographic spinal progression in the logistic regression analysis (Table 5). Similar to the data presented in Table 4, the association between serum adipokines levels and radiographic spinal progression was evident in men but not in women when analyzed separately (Table 6).

**Table 5:** Analysis of the association of leptin and HMW-APN with radiographic spinal progression after 2 years

	OR unadjusted	95% CI	OR adjusted^a^	95% CI
*Outcome: no mSASSS progression after 2 years (logistic regression)*				
Leptin, ng/mL	1.06	1.003 to 1.12	1.16	1.03 to 1.29
Leptin/BMI	9.20	1.44 to 58.7	28.7	2.24 to 367.7
HMW-APN, μg/mL	1.12	0.98 to 1.27	1.17	0.99 to 1.38
HMW-APN/BMI	9.68	0.48 to 196.2	10.7	0.35 to 320.6
HMW-APN/APN	10.81	1.25 to 93.5	22.2	1.57 to 313.1
*Outcome: no syndesmophyte formation/progression after 2 years (logistic regression)*				
Leptin, ng/mL	1.07	1.002 to 1.14	1.29	1.11 to 1.50
Leptin/BMI	36.5	3.29 to 404.5	131.9	4.78 to 3638
HMW-APN, μg/mL	1.18	1.01 to 1.39	1.18	0.98 to 1.42
HMW-APN/BMI	34.3	0.89 to 1319	23.8	0.41 to 1382
HMW-APN/APN	10.9	1.11 to 106.3	10.8	0.74 to 156.1

^a^Adjusted for the presence of syndesmophytes at baseline, C-reactive protein at baseline, sex, non-steroidal anti-inflammatory drugs intake score over 2 years, and smoking status at baseline. Models with leptin, HMW-APN and HMW-APN/APN ratio were additionally adjusted for body mass index (BMI). *APN* adiponectin, *CI* confidence interval, *HMW-APN* high molecular weight adiponectin, *mSASSS* modified Stoke Ankylosing Spondylitis Spine Score, *OR* odds ratio

**Table 6:** Analysis of association of leptin and HMW-APN with radiographic spinal progression stratified by sex

	OR unadjusted	95% CI	OR adjusted^a^	95% CI
Men (n = 82)				
*Outcome: no mSASSS progression ≥2 points after 2 years (logistic regression)*				
Leptin, ng/mL	1.20	1.06 to 1.36	1.45	1.18 to 1.78
Leptin/BMI	725.2	15.4 to 34099	4818.9	33.3 to 698123
HMW-APN μg/mL	1.25	1.002 to 1.57	1.35	1.04 to 1.75
HMW-APN/BMI	81.8	0.53 to 12683	186.4	0.63 to 54915
HMW-APN/APN	40.5	2.01 to 813.9	173.6	4.03 to 7479
*Outcome: no syndesmophyte formation/progression after 2 years (logistic regression)*				
Leptin, ng/mL	1.06	0.97 to 1.16	1.34	1.11 to 1.62
Leptin/BMI	54.5	1.99 to 1491	241.8	3.51 to 16651
HMW-APN, μg/mL	1.20	0.97 to 1.48	1.21	0.95 to 1.53
HMW-APN/BMI	55.3	0.37 to 8262	77.9	0.32 to 19249
HMW-APN/APN	31.5	1.60 to 621.5	55.6	1.56 to 1973
Women (n = 38)				
*Outcome: no mSASSS progression ≥2 points after 2 years (logistic regression)*				
Leptin, ng/mL	1.01	0.96 to 1.07	1.04	0.95 to 1.14
Leptin/BMI	1.53	0.21 to 11.29	1.67	0.15 to 18.8
HMW-APN, μg/mL	0.99	0.85 to 1.17	1.04	0.85 to 1.27
HMW-APN/BMI	1.22	0.03 to 48.9	3.03	0.05 to 196.9
HMW-APN/APN	0.69	0.02 to 30.4	0.47	0.003 to 68.6
*Outcome: no syndesmophyte formation/progression after 2 years (logistic regression)*				
Leptin, ng/mL	1.05	0.95 to 1.16	1.17	0.90 to 1.51
Leptin/BMI	20.5	0.23 to 1872	18.7	0.06 to 5989
HMW-APN, μg/mL	1.07	0.83 to 1.38	1.13	0.84 to 1.51
HMW-APN/BMI	3.48	0.02 to 738.3	14.8	0.03 to 7869
HMW-APN/APN	0.15	0.001 to 21.4	0.15	0.0004 to 59.5

^a^Adjusted for the presence of syndesmophytes at baseline, C-reactive protein at baseline, non-steroidal anti-inflammatory drugs intake score over two years, and smoking status at baseline. Models with leptin, high molecular weight adiponectin (HMW-APN) and HMW-APN/adiponectin (APN) ratio were additionally adjusted for body mass index (BMI). *CI* confidence interval, *mSASSS* modified Stoke Ankylosing Spondylitis Spine Score, *OR* odds ratio

## Discussion

In the current study we investigated the association between serum adipokines and radiographic spinal progression in patients with AS. We report for the first time that baseline leptin and HMW-APN serum levels, and to a further extent the HMW-APN/APN ratio were inversely associated with (i.e., predicted protection against) radiographic spinal progression. Initially, we investigated eight adipokines and only those with evidence suggesting an association with radiographic progression were entered the main analysis. We have chosen the homogeneous population of the ENRADAS trial consisting of patients with advanced AS. The lack of differences in radiographic spinal progression between the study arms justified pooling both groups of patients (treated with NSAIDs continuously or on demand). However, due to some change in the leptin level over 2 years that was especially evident in the continuous treatment group, the parameter estimates in the regression analysis were adjusted for NSAID intake.

In the study population, leptin and HMW-APN levels were higher in female patients with AS compared to male patients, even after adjustment for BMI. At the same time, there was no association between BMI and radiographic spinal progression. There are data on gender-dependent differences in adipokine levels in the general population [29, 30]. In one study, leptin and APN levels were higher in women than in men independent of BMI or waist-to-hip ratio [29], while in another study such an association was shown for leptin, but not for adiponectin [30]. Similarly, differences in adipokine levels between male and female patients have also been described in patients with AS [17, 31]. Such an association between sex and adipokine levels is especially interesting in the context of the current study, since the women in our study also had significantly lower mSASSS scores than men. It has been shown several times that female patients with AS develop new bone formation to a lesser extent than male patients with AS [5, 20]. Therefore, we can speculate that leptin and HMW-APN might be biomarkers (or even pathophysiological factors) linking gender with radiographic spinal progression in patients with AS.

Remarkably, in the analyses stratified by sex, an association between adipokines and radiographic spinal progression was evident in male patients only. On the one hand, the absence of association between serum adipokine and radiographic spinal progression in female patients could be related to the smaller sample size and a small number of progressors in the female group. On the other hand, female patients had generally higher levels of leptin and adiponectin in comparison to male patients, suggesting that in female patients who progressed after 2 years, there could have been other factors playing a part.

In our previous study involving patients from the German Spondyloarthropathy Inception Cohort (GESPIC) cohort, who had early axial SpA, we found a positive association between visfatin and radiographic spinal progression; leptin and HMW-APN were not investigated [17]. In the current study, we found only a trend for such an association for visfatin that might be related to the differences in disease duration in the GESPIC (early disease) and ENRADAS (rather advanced disease) studies or to the test system used.

Until now, serum adipokines and their relationship to clinical or radiographic parameters in AS have only been investigated in a few small trials. In some studies, leptin serum was found to be lower in patients with AS compared to healthy controls [32–34], but in others there were either no differences or even higher leptin serum levels in patients with AS [35–37]. A meta-analysis from Mei et al, which included eight studies that had compared leptin levels in patients with AS with those in healthy controls, came to the conclusion that there are no significant differences in leptin levels in patients with AS vs. controls [38]. Differences in serum APN (including the HMW isoform) have also not been identified at group level in patients with AS and healthy controls [17, 32, 34]. However, in one study serum APN was higher in male patients with AS versus healthy controls, and at the same time there was almost no correlation between leptin or APN and inflammatory activity, and anti-inflammatory treatment (anti-TNF-α) did not have an effect on serum leptin and APN [35].

Data on the association between structural damage in the spine and leptin in AS are very limited. In one study, higher serum leptin was positively associated with the presence of syndesmophytes in a cross-sectional analysis; however, no prospective data were reported [36]. In the aforementioned study of patients with early axial SpA, APN was only numerically higher in non-progressors, but the HMW isoform was not investigated [17].

What are the possible mechanisms linking leptin and adiponectin with radiographic spinal progression in AS? Adipokines are known to be involved in the regulation of immune processes, displaying both pro-inflammatory and anti-inflammatory effects, and in bone metabolism that explains their possible relationship with the pathophysiology of chronic inflammatory conditions such as rheumatoid arthritis or AS [21, 22, 39]. Our current observation of a protective effect of leptin and APN (especially its HMW isoform) against radiographic spinal progression in AS can probably be best explained by the effects of both molecules on the process of osteogenesis, since there is no association between adipokines and inflammatory activity as measured by CRP.

Many studies have previously assessed the part played by leptin in bone metabolism; however, published data show both positive and negative effects of leptin on bone formation [40]. Two main regulatory pathways are currently discussed. One targets the central nervous system, the other one acts through peripheral regulation [41]. The peripheral effect is based on a rather direct interaction of leptin with several bone cell types. For instance, leptin enhances new bone formation through interaction with bone marrow stem cells, which can either differentiate into osteoblasts or into adipocytes. It promotes differentiation and growth of osteoblasts and enhances bone mineralization [42–44]. Furthermore, leptin administered peripherally can inhibit formation of human osteoclasts [44, 45].

In contrast, a central regulatory effect of leptin on bone metabolism is rather anti-osteoproliferative. It has been shown that bone loss could be induced in leptin-deficient mice through an intracerebrovascular infusion of leptin [46], indicating a role of leptin as a hormone which induces pathways of the sympathetic nervous system (SNS) through the ventromedial hypothalamus (VMH), which then results in bone loss. Further studies have confirmed this theory [47, 48]. The available evidence indicates that leptin binds to its receptors on neurons of the VMH, which then activate a signal pathway from the VMH to osteoblasts via β2 adrenergic receptors of the SNS. Through the signals of the SNS two cascades in osteoblasts are activated leading to increased osteoclast formation and decreased osteoblast differentiation [41].

Hamrick et al. proved in 2004 that the effects of leptin on the skeleton differ between skeletal regions. Comparing leptin-deficient obese mice (ob/ob) with lean mice they found that ob/ob mice had significantly reduced femoral bone mineral density (BMD), bone mineral content (BMC), cortical thickness and trabecular bone volume compared to lean mice. On the other hand, they found ob/ob mice to have higher BMC, BMD and trabecular bone volume in the lumbar spine than the lean mice. Hence, their study suggests that leptin regulates bone metabolism differently according to the location within the skeleton [49]. Martin et al. found in 2007 that there could be a bimodal threshold response to serum leptin levels, meaning that slight increases in leptin might initially stimulate bone formation, while higher leptin levels inhibit bone formation [50].

Thus, leptin might have contradictory effects on bone metabolism that also depend on the localization of the bone. We hypothesize that in patients with AS a higher level of leptin has protective properties against radiographic spinal progression mainly via the central pathway with inhibition of excessive new bone formation.

As for APN, different effects on bone metabolism have been discovered as well, yielding similarly conflicting results. APN was found to be expressed in osteoblasts along with its receptors and to stimulate their proliferation and differentiation, for example, through enhancement of the expression of bone morphogenetic protein 2 (BMP-2) [51–53]. Additionally, APN was found to be able to suppress osteoclastogenesis and promote osteoblastogenesis [54]. However, APN is also able to indirectly activate osteoclasts by stimulating receptor activator of nuclear factor kappa-B (NFкB) ligand (RANKL) and inhibiting osteoprotegerin (OPG) production in osteoblasts, which are known to be essential stimulators of osteoclastogenesis [55].

An inverse relationship between APN and BMD was found in vivo in a number of studies [39] indicating that APN might have predominantly catabolic effects on bone. In line with this, the meta-analysis of Biver et al., which included 59 studies of healthy male and female patients who were evaluated for BMD or fracture risk and adipokines, showed that APN seems to be the most important adipokine that is negatively associated with BMD [56].

There are two main circulating isoforms of APN in the blood stream: one low molecular weight (LMW) hexamer and the HMW multimer [28, 57]. Previous data suggest that HMW-APN might be the predominantly active isoform and that the HMW-APN/APN ratio might be an important determinant of the metabolic effects of the hormone [28]. The importance of the HMW-APN/APN ratio was also confirmed in another study, in which the ratio was a better predictor of insulin resistance and metabolic syndrome than total plasma APN [58]. In line with these data, we found that HMW-APN (and to a further extent the HMW-APN/APN ratio) was inversely associated with radiographic spinal progression in AS, and the association between radiographic progression and HMW-APN was better than that between radiographic progression and total APN.

Our study has limitations. First, this was an analysis based on a population of a randomized controlled trial. Although we corrected our analysis for differences in the intervention, we cannot completely exclude bias resulting from treatment. Further, only relatively few women were included in this trial, which made the interpretation of the subgroup analysis results difficult in this population. Also, no fat mass measurement was performed in the ENRADAS trial beyond BMI calculation. Finally, since no biologic agents were allowed in the ENRADAS trial it is currently unclear whether leptin and HMW-APN might also have protective effects against radiographic spinal progression in patients treated with anti-TNF or anti-interleukin-17 drugs. Therefore, an independent validation of our results is highly desirable not only in patients treated with NSAIDs but also in patients undergoing biologic treatment. Despite a significant association with radiographic spinal progression, leptin or HMW-APN alone would not be sufficient for identification of patients at high (or low) risk of radiographic spinal progression. However, these biomarkers seem to be good candidates for inclusion in multi-parameter predictive models for estimation of the risk of radiographic spinal progression in AS.

## Conclusion

Serum levels of leptin and HMW-APN are inversely associated with radiographic spinal progression in patients with AS. Current data on the role of adipokines in bone metabolism indicates that leptin and HMW-APN might not only be markers but also true pathophysiological factors influencing new bone formation in AS. Female patients with AS have a higher level of leptin and APN, which might play a natural protective role against radiographic spinal progression in AS, explaining the generally lower prevalence of extended new bone formation in the spine in female patients with AS.

## Abbreviations

ANCOVA: Analysis of covariance
APN: Adiponectin
AS: Ankylosing spondylitis
ASDAS: Ankylosing Spondylitis Disease Activity Score
BMC: Bone mineral content
BMD: Bone mineral density
BMI: Body mass index
BMP-2: Bone morphogenetic protein 2
CRP: C-reactive protein
ELISA: Enzyme-linked immunosorbent assays
ENRADAS: Effects of NSAIDs on radiographic damage in AS
GESPIC: German spondyloarthropathy inception cohort
HMW-APN: High molecular weight adiponectin
LMW: Low molecular weight
MRI: Magnetic resonance imaging
mSASSS: Modified Stoke Ankylosing Spondylitis spine score
NFкB: Nuclear factor kappa B
NSAIDs: Non-steroidal anti-inflammatory drugs
OPG: Osteoprotegerin
RANKL: Receptor activator of NFкB ligand
ROC: Receiver operating characteristic
SNS: Sympathetic nervous system
SpA: Spondyloarthritides
TNF: Tumor necrosis factor
VMH: Ventromedial hypothalamus
